# Parenthood and the risk of diabetes in men and women: a 7 year prospective study of 0.5 million individuals

**DOI:** 10.1007/s00125-016-3980-x

**Published:** 2016-05-18

**Authors:** Sanne A. E. Peters, Ling Yang, Yu Guo, Yiping Chen, Zheng Bian, Iona Y. Millwood, Fiona Bragg, Xue Zhou, Pengfei Ge, Biyun Chen, Yulian Gao, Yijun Li, Junshi Chen, Liming Li, Mark Woodward, Zhengming Chen, on behalf of the China Kadoorie Biobank Collaboration Group

**Affiliations:** The George Institute for Global Health, University of Oxford, 34 Broad Street, Oxford, OX1 3BD UK; Clinical Trial Service Unit and Epidemiological Studies Unit, University of Oxford, Old Road Campus, Oxford, OX3 7LF UK; Chinese Academy of Medical Sciences, Dong Cheng District, Beijing, People’s Republic of China; Department of Prevention and Control of Non-Communicable Diseases, Heilongjiang Center for Disease Control and Prevention, Harbin, Heilongjiang People’s Republic of China; Gansu Center for Disease Control and Prevention, Lanzhou, Gansu People’s Republic of China; Department of Prevention and Control of Non-Communicable Diseases, Hunan Center for Disease Control and Prevention, Hunan Changsha, People’s Republic of China; Huixian Center for Disease Control and Prevention, Huixian, Henan People’s Republic of China; Department of Prevention and Control of Non-Communicable Diseases, Meilan Center for Disease Control and Prevention, Haikou, Hainan People’s Republic of China; China National Center for Food Safety Risk Assessment, Chaoyang District, Beijing, People’s Republic of China; Department of Public Health, Beijing University, Beijing, People’s Republic of China; The George Institute for Global Health, University of Sydney, Sydney, NSW Australia; Department of Epidemiology, Johns Hopkins University, Baltimore, MD USA

**Keywords:** Children, China, Diabetes, Men, Parenthood, Women

## Abstract

**Aims/hypothesis:**

In women, higher parity has been associated with increased risk of diabetes later in life. It is unclear, however, whether this association is mainly due to biological effects of childbearing, or to socioeconomic and lifestyle factors associated with childrearing. We assessed the association between number of children and diabetes risk separately in women and men.

**Methods:**

Between 2004 and 2008, the nationwide China Kadoorie Biobank recruited 0.5 million individuals aged 30–79 (mean 51 years) from ten diverse regions across China. During 7 years of follow-up, 8,840 incident cases of diabetes were recorded among 463,347 participants without prior cardiovascular diseases or diabetes. Multivariable Cox regression yielded sex-specific HRs and 95% CIs for incident diabetes by number of children.

**Results:**

Overall, ∼98% of all participants had children. In women, there was a J-shaped association between number of children and risk of diabetes. Compared with women with one child, the adjusted HRs for diabetes were 1.39 (95% CI 1.11, 1.73) for childless women, 1.12 (95% CI 1.07, 1.18) for those with two children, 1.23 (95% CI 1.16, 1.31) for those with three children, and 1.32 (95% CI 1.21, 1.44) for those with four or more children. In men, there was a similar association with risk of diabetes; the corresponding HRs were 1.28 (95% CI 1.02, 1.60), 1.19 (95% CI 1.12, 1.26), 1.32 (95% CI 1.21, 1.44) and 1.41 (95% CI 1.24, 1.60), respectively. In both sexes, the findings were broadly similar in different population subgroups.

**Conclusions/interpretation:**

The similarity between women and men in the association between number of children and risk of diabetes suggests that parenthood is most likely to affect diabetes risk through factors associated with childrearing rather than via biological effects of childbearing.

**Electronic supplementary material:**

The online version of this article (doi:10.1007/s00125-016-3980-x) contains peer-reviewed but unedited supplementary material, which is available to authorised users.

## Introduction

Pregnancy causes marked alterations in women’s metabolic profile, including reduced insulin sensitivity, increased production of insulin, and accumulation and redistribution of body fat. These changes could lead to gestational diabetes and may also increase the mother’s risk of developing diabetes and associated cardiometabolic diseases later in life [1–3]. Several studies of mostly Western populations have examined the association between parity and risk of diabetes later in life, but have had inconclusive findings. A number of studies found that parity, particularly grand multiparity, is associated with an increased risk of diabetes [4–11], but this was not supported by other studies [12–14]. This discordance between different studies has prompted the discussion as to whether the association between parity and diabetes risk reflects metabolic consequences of pregnancy and childbearing itself, or whether socioeconomic and lifestyle factors associated with childrearing underpin this association [15–17]. Studies attempting to address this issue have generated inconsistent results: some studies reported that most of the association between parity and diabetes was mediated by socioeconomic factors and BMI [12, 14], while others showed that the relationship remained after allowing for these factors [5–8, 10, 11].

Examination of the relationship between parenthood and the risk of diabetes in men provides a useful insight into the relative importance of the biological factors related to pregnancy, and the socioeconomic and lifestyle factors related to childrearing, especially when combined with simultaneous examination in women from the same study. Studies have used this approach to examine the effects of having children on cardiovascular diseases [16, 18], yet no such male and female comparative studies have been reported on diabetes. Moreover, the majority of studies assessing the relationship between parity and the onset of diabetes have involved Western populations. Of particular interest are the effects of parity on the development of diabetes in China, a population where reproductive patterns are changing, especially following the introduction of the one-child policy during the early 1980s, yet are still importantly different from those in Western countries [19].

We examined the relationship between parenthood and the risk of incident diabetes in both women and men in the China Kadoorie Biobank (CKB) [20], a prospective study of 500,000 individuals recruited from ten diverse regions in China.

## Methods

### Baseline survey

Detailed information about the design and study procedures of the CKB has been reported previously [20]. In brief, the baseline survey took place from June 2004 to July 2008 in ten geographically defined areas of China, and a total of 512,891 individuals (59% women) were enrolled. At the baseline survey, information about demographic and socioeconomic status (e.g. education, marital status, number of ever-born biological children), lifestyle factors (e.g. smoking, physical activity), personal and family medical history, and women’s reproductive history were collected using an interviewer-administered laptop-based questionnaire, along with a range of physical measurements. A blood sample was collected from each participant for an immediate on-site test of blood glucose using the SureStep Plus meter (LifeScan, Milpitas, CA, USA) and long-term storage. Following completion of the baseline survey, two resurveys of 5–6% randomly selected surviving participants were undertaken, using procedures similar to those at study baseline [20]. The *κ* value for repeatability was 0.93, comparing the reported number of children at study baseline and at the first resurvey, indicating high agreement. Central ethics approvals were obtained from the University of Oxford, the China National Center for Disease Control and Prevention (CDC), and the institutional research boards at the local CDCs in the ten regions.

### Follow-up and endpoint definition

Since recruitment, study participants have been followed up for cause-specific morbidity and mortality through linkage with regional disease and death registers and with the recently established national health insurance system [20]. Causes of death are sought chiefly from official death certificates and are, where necessary, supplemented by reviews of medical records. Data linkage with health insurance agencies is carried out every 6 months in each region, and all hospitalised events occurring in that last half-year are retrieved for matched study participants. At present, ∼98% of the study population is covered by the health insurance system. Active follow-up is performed on an annual basis to minimise losses to follow-up.

For the present study, the primary endpoint was incident diabetes mellitus, as defined by codes E10–E14 in the tenth edition of the International Classification of Diseases (ICD-10) www.who.int/classifications/icd/en/. A separate outcome adjudication study during 2012–2013, involving careful review of the original medical records and laboratory tests in ∼1,000 randomly selected diabetes cases, showed that the diagnoses of diabetes in CKB were of extremely high quality, with almost 99% confirmed by an independent expert panel.

Individuals (*n* = 23,129 [57% women]) with a self-reported history of cardiovascular diseases were excluded from the present analyses. Also excluded were individuals with a self-reported history of diabetes (*n* = 13,313 [62% women]) or screen-detected diabetes (*n* = 13,102 [61% women]), defined as no self-reported diabetes and a blood glucose level ≥7.0 mmol/l and a fasting time >8 h, a blood glucose level ≥11.1 mmol/l and a fasting time <8 h (nine study areas), or a fasting blood glucose level ≥7.0 mmol/l (one study area). After these exclusions, 463,347 (189,964 men and 273,383 women) remained for the final analyses.

### Statistical analyses

Baseline characteristics are presented as means (SD) for continuous variables and as percentages for categorical variables. Cox proportional hazards models were used to estimate sex-specific HRs and 95% CIs for incident diabetes by the number of children, categorised as no children, one child (reference), or two, three, or four or more children. CIs were estimated using the floating absolute risks method [21]. We also obtained the HRs and CIs for childless individuals compared with individuals with children. In analyses restricted to individuals who had at least one child, we estimated the HRs and CIs per additional child. Analyses were stratified by age at risk and area of residence (model I), and were additionally adjusted for level of attained education and household income (model II), followed by further adjustment for smoking status, alcohol use, physical activity, systolic blood pressure, history of hypertension and BMI (model III).

Predefined subgroup analyses were conducted to obtain the HRs and CIs for incident diabetes per additional child by study region, age group, highest level of attained education, BMI, smoking status and history of hypertension. Three sensitivity analyses were conducted. First, to assess the impact of clustering of risk factors on our results, we excluded individuals whose partner had also contributed to the study. Matching was done based on name, address and telephone number. Second, to determine the impact of individuals with unusually large family sizes, we excluded those who had more than ten children. Third, we examined the associations of diabetes with parity (i.e. the number of live births) in women. Analyses were performed using SAS version 9.3 (SAS Institute, Cary, NC, USA) and R version 3.1.2 (R Foundation for Statistical Computing, Vienna, Austria).

## Results

Of the 463,347 participants included, the mean baseline age was 51 (SD 11) years, and 59% were women. More than 98% of women and 97% of men had children, with about two-thirds having one or two children (Table 1 for women and Table 2 for men). In both sexes, individuals with one child were generally younger, better educated and had a higher household income compared with individuals with no children or with more than one child. The prevalence of current smoking and weekly alcohol use was considerably higher in men than in women across all parenthood categories. Systolic blood pressure and the prevalence of hypertension were lowest in individuals with one child. Stillbirth and abortion were considerably more common in women without children than in those with children.

**Table 1:** Baseline characteristics of women by number of children

Characteristic	Total	No children	One child	Two children	Three children	Four or more children
*N* (%)	273,383	3,765 (1.3)	99,859 (35.5)	90,505 (32.2)	46,965 (16.7)	32,289 (11.5)
Rural, %	57.2	37.0	35.8	70.3	70.8	69.0
Age, years	50.1 (10.3)	48.8 (11.7)	44.4 (6.8)	49.0 (8.8)	55.8 (9.3)	63.0 (8.6)
Education level, %						
Primary or below	56.3	39.0	31.3	61.6	77.6	89.9
Secondary or above	43.7	61.0	68.7	38.4	22.4	10.1
Married, %	89.8	69.6	93.7	93.5	86.5	73.9
Household income, %						
Low	10.2	11.4	4.6	7.9	14.9	26.4
Middle	49.2	51.3	42.5	51.1	55.8	54.3
High	40.7	37.3	52.8	41.0	29.2	19.3
Current smoking, %	2.2	3.6	1.7	1.6	2.8	4.7
Weekly alcohol use, %	2.1	3.1	2.6	1.6	1.9	2.4
Physical activity, MET h/day	17.7 (11.2, 29.1)	15.2 (9.5, 25.4)	20.2 (13.0, 31.6)	18.2 (11.2, 30.4)	15.2 (10.3, 26.5)	11.7 (8.4, 20.6)
Systolic blood pressure, mmHg	128.7 (21.5)	125.5 (22.7)	122.0 (18.0)	128.8 (20.6)	134.7 (22.6)	140.3 (24.2)
BMI, kg/m^2^	23.7 (3.4)	23.2 (3.7)	23.7 (3.2)	23.8 (3.4)	23.7 (3.5)	23.4 (3.7)
History of hypertension, %	9.2	8.0	5.5	8.9	13.1	16.1
Stillbirth and abortion, %						
History of stillbirth	6.4	71.7	2.3	5.2	8.1	12.3
History of induced abortion	52.7	88.4	70.7	47.1	37.0	31.1
History of spontaneous abortion	9.8	75.1	5.0	9.0	11.8	16.0

Values are percentages for categorical variables, and means (SD) or median (25th and 75th percentiles) for continuous variables

MET, metabolic equivalent

**Table 2:** Baseline characteristics of men by number of children

Characteristic	Total	No children	One child	Two children	Three children	Four or more children
*N* (%)	189,964	5,747 (2.9)	72,224 (37.0)	60,872 (31.2)	31,341 (16.1)	19,780 (10.1)
Rural, %	58.5	62.3	36.7	71.6	72.3	74.0
Age, years	51.6 (10.8)	49.0 (12.1)	45.6 (7.7)	51.4 (9.6)	58.2 (9.4)	64.5 (8.1)
Education level, %						
Primary or below	42.5	55.1	25.1	46.4	56.0	69.3
Secondary or above	57.5	44.9	74.9	53.6	44.0	30.7
Married, %	93.0	38.4	95.5	96.1	93.8	88.7
Household income, %						
Low	9.3	30.3	4.1	6.9	13.0	23.7
Middle	45.3	42.8	36.8	47.7	54.5	55.0
High	45.4	26.9	59.1	45.3	32.6	21.4
Current smoking, %	62.5	60.3	64.0	63.2	61.0	57.9
Weekly alcohol use, %	34.0	27.0	42.7	31.4	27.2	23.1
Physical activity, MET h/day	19.9 (10.7, 33.3)	18.5 (11.1, 31.0)	23.5 (14.2, 34.5)	21.4 (10.8, 36.5)	16.1 (7.8, 29.3)	11.6 (5.6, 22.6)
Systolic blood pressure, mmHg	131.9 (19.7)	131.4 (20.4)	129.0 (17.4)	131.7 (19.4)	134.9 (21.2)	138.7 (22.7)
BMI, kg/m^2^	23.3 (3.2)	22.5 (3.3)	23.9 (3.2)	23.2 (3.1)	22.8 (3.1)	22.5 (3.2)
History of hypertension, %	8.8	5.6	6.2	8.8	12.1	14.2

Values are percentages for categorical variables, and means (SD) or median (25th and 75th percentiles) for continuous variables

MET, metabolic equivalent

**Fig. 1:**
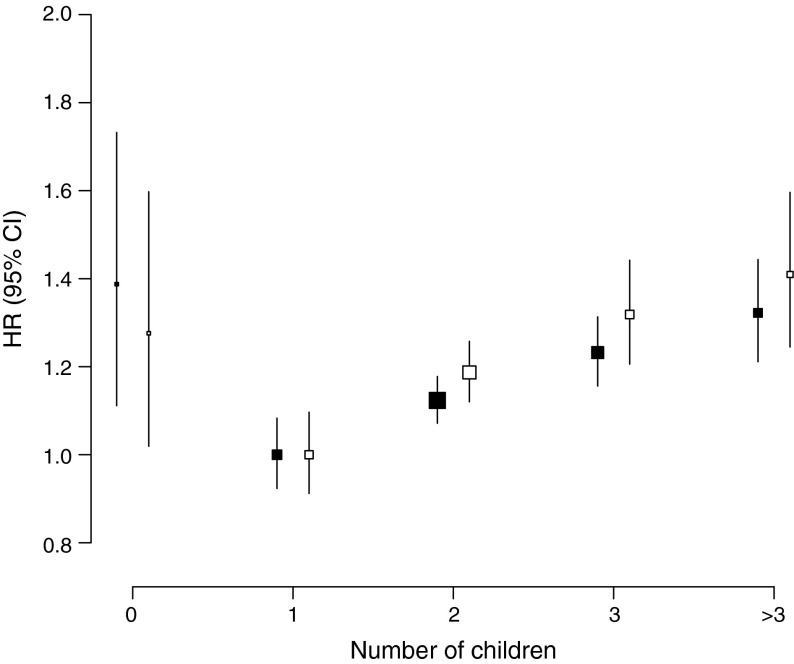
Adjusted HRs for incident diabetes associated with number of children. Analyses are stratified by age and region, and additionally adjusted for level of attained education, household income, smoking status, alcohol use, systolic blood pressure, history of hypertension, physical activity and BMI. The HRs are plotted on a floating absolute scale. Each square has an area inversely proportional to the standard error of the natural log risk. Vertical lines indicate the corresponding 95% CIs. Black squares, women (5,579 events); white squares, men (3,261 events)

**Fig. 2:**
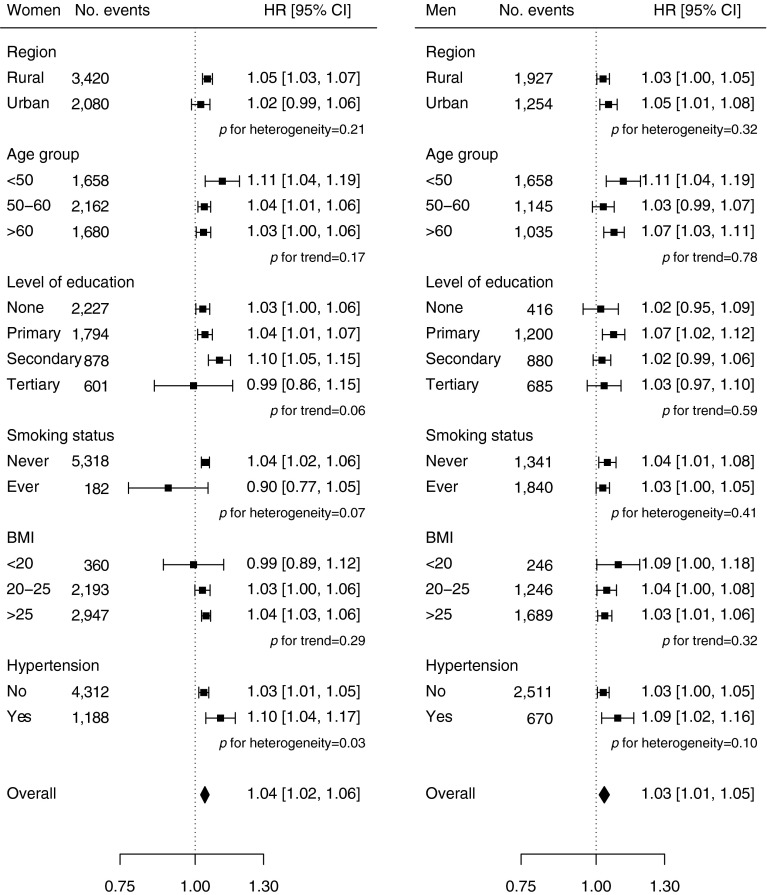
Adjusted HRs for incident diabetes per additional child by baseline characteristics in women and men. Analyses are stratified by age and region, and adjusted for level of attained education, household income, smoking status, alcohol use, systolic blood pressure, history of hypertension, physical activity and BMI. Each square represents the risk of diabetes per additional child, with its area inversely proportional to the standard error of the log risk. The diamond indicates the overall diabetes risk per additional child and the 95% CI. Individuals without children are excluded

**Fig. 3:**
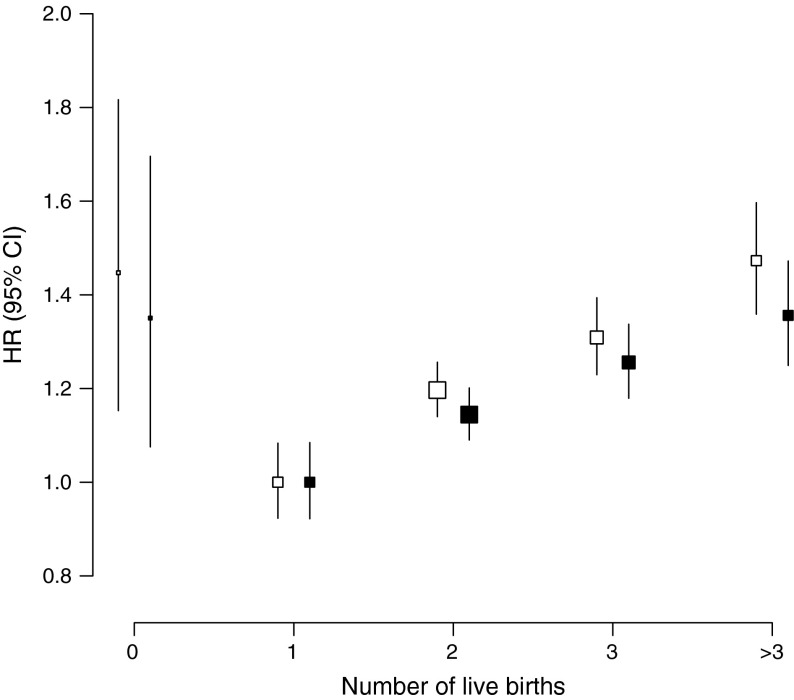
Adjusted HRs for incident diabetes associated with number of live births. Analyses are stratified by age and region (white squares, model I), and additionally adjusted for level of attained education, household income, smoking status, alcohol use, systolic blood pressure, history of hypertension, physical activity and BMI (black squares, model III). Conventions are the same as in Fig. 1

During a median of 7.2 years (interquartile range 6.3–8.1) of follow-up, 8,840 (5,579 women and 3,261 men) incident cases of diabetes were recorded. Overall, childless women had a 27% (adjusted HR 1.27 [95% CI 1.01, 1.59]) excess risk of diabetes compared with women with children in the age- and region-stratified model, which attenuated slightly (HR 1.22 [95% CI 0.97, 1.52]) after further adjustment for socioeconomic and lifestyle factors (Table 3). In women with at least one child, there was a log-linear association between the number of children and the risk of diabetes (Table 3, Fig. 1). In analyses stratified by age and region, women with two, three, or four or more children were at a 17%, 30% and 45% increased risk of diabetes compared with women with one child (*p* < 0.001 for trend). Further adjustment for socioeconomic and lifestyle factors only slightly attenuated the relationship, with adjusted HRs of 1.12 (95% CI 1.07, 1.18), 1.23 (95% CI 1.16, 1.31) and 1.32 (95% CI 1.21, 1.44), respectively, for two, three and four or more children (*p* < 0.001 for trend). In analyses restricted to women with children, each additional child was associated with a 4% increased risk of diabetes (HR 1.04 [95% CI 1.02, 1.06]), rising to 1.06 (95% CI 1.03, 1.09) in analyses restricted to women with up to ten children (electronic supplementary material [ESM] Table 1), with limited evidence for material differences between population subgroups (Fig. 2). However, there was some indication that the magnitude of the effect was stronger in women with a history of hypertension compared with those without (*p* = 0.03 for heterogeneity). Findings for the association between the number of live births and the risk of diabetes were similar (Fig. 3).

**Table 3:** HRs (95% CIs) for incident diabetes associated with number of children

	Childless vs not	Number of children					Per additional child^a^
		No children	One child	Two children	Three children	Four or more children	
Women							
No. of events		79	1,417	1,978	1,209	896	
Model I	1.27 (1.01, 1.59)	1.50 (1.20, 1.87)	1.00 (0.92, 1.08)	1.17 (1.12, 1.23)	1.30 (1.22, 1.38)	1.45 (1.33, 1.58)	1.05 (1.03, 1.06)
Model II	1.28 (1.03, 1.61)	1.50 (1.20, 1.87)	1.00 (0.92, 1.08)	1.16 (1.10, 1.21)	1.27 (1.19, 1.36)	1.42 (1.30, 1.55)	1.04 (1.03, 1.05)
Model III	1.22 (0.97, 1.52)	1.39 (1.11, 1.73)	1.00 (0.92, 1.08)	1.12 (1.07, 1.18)	1.23 (1.16, 1.31)	1.32 (1.21, 1.44)	1.04 (1.02, 1.06)
Men							
No. of events		80	1,012	1,177	618	374	
Model I	0.96 (0.76, 1.20)	1.12 (0.90, 1.39)	1.00 (0.91, 1.10)	1.18 (1.12, 1.25)	1.30 (1.19, 1.43)	1.42 (1.25, 1.60)	1.04 (1.02, 1.06)
Model II	1.03 (0.82, 1.29)	1.22 (0.97, 1.53)	1.00 (0.91, 1.10)	1.20 (1.13, 1.27)	1.34 (1.23, 1.47)	1.50 (1.32, 1.69)	1.04 (1.02, 1.06)
Model III	1.09 (0.87, 1.37)	1.28 (1.02, 1.60)	1.00 (0.91, 1.10)	1.19 (1.12, 1.26)	1.32 (1.21, 1.44)	1.41 (1.24, 1.60)	1.03 (1.01, 1.05)

Model I, age and region; model II, model I + level of attained education and household income; model III, model II + smoking status, alcohol use, systolic blood pressure, history of hypertension, physical activity and BMI

^a^Analyses restricted to individuals with children

Among men, the association between number of children and diabetes was similar to that in women. Compared with men without children, the HRs of diabetes for men with children were 0.96 (95% CI 0.76, 1.20) in the age- and region-stratified models, and 1.09 (95% CI 0.87, 1.37) after additional adjustment for socioeconomic and lifestyle factors (Table 3). In men with children, there was a positive association between the number of children and the risk of diabetes. In analyses stratified by age and region, and adjusted for socioeconomic and lifestyle factors, HRs for diabetes were 1.00 (95% CI 0.91, 1.10), 1.19 (95% CI 1.12, 1.26), 1.32 (95% CI 1.21, 1.44) and 1.41 (95% CI 1.24, 1.60) for one, two, three and four or more children (*p* < 0.001 for trend). Each additional child was associated with an HR of 1.03 (95% CI 1.01, 1.05) for diabetes (1.09 [95% CI 1.05, 1.14] in analyses restricted to men with up to ten children [ESM Table 1]), with little evidence of significant differences between subgroups (Fig. 2).

The fully adjusted HR of diabetes comparing those without children with those with children was slightly more extreme in women than in men (1.22 [95% CI 0.97, 1.52] vs 1.09 [95% CI 0.87, 1.37]), but the difference was not significant (*p* = 0.63 for heterogeneity). Nor was there any heterogeneity between men and women in the relationship between each additional child and the risk of diabetes among those with children (*p* = 0.51 for heterogeneity). Excluding individuals whose partner had also participated in the study yielded similar results (1.03 [95% CI 1.01, 1.05] vs 1.03 [95% CI 1.01, 1.05]; *p* = 0.53 for heterogeneity [ESM Table 1]).

## Discussion

This large prospective study of half a million individuals in China provides the most robust evidence to date on the relationship between having children and the risk of diabetes in later life. In both women and men, individuals without children or with multiple children were at a higher risk of diabetes compared with those with only one child. Among individuals with children, each additional child was associated with a 3–4% increased risk of diabetes in both women and men. The similarity of the association in women and men suggests that factors associated with childrearing rather than biological effects of childbearing are most likely to be underpinning the increased risk of diabetes in women. Previous studies on the association between parity and risk of diabetes in women have reported discordant results [4–14]. A Danish study of 100,669 women identified through the National Birth Registry showed that the risk of diabetes increased with higher parity, primarily among younger women [4]. These analyses, however, were not adjusted for BMI and other socioeconomic factors, which were found to be important confounders or mediators in other studies. For example, the Nurses’ Health Study, which included 113,606 women and 2,310 cases of diabetes, did observe an association between parity and incident diabetes before, but not after, adjustment for BMI [14]. The Atherosclerosis Risk in Communities (ARIC) study of 7,024 women and 754 cases of diabetes also demonstrated that much of the risk of diabetes associated with parity disappeared after adjustment for obesity [5]. However, grand multiparity (i.e. five or more live births) was still associated with a 27% (95% CI 2%, 57%) increased risk of diabetes, after adjustment for these factors [5]. Similarly, the Singapore Chinese Health Study among 25,021 Chinese women and 1,294 cases of diabetes showed that women with three or more live births had a 60% excess risk of diabetes compared with nulliparous women [6], after adjustment for a range of demographic, lifestyle and reproductive health factors, including BMI. The present analyses, which included over 5,500 incident cases of diabetes in 273,383 women, suggest that the association between number of children and diabetes in women persisted, though attenuated slightly, after controlling for a range of potential confounders and mediators.

It has been proposed that several pregnancy-induced physiological changes may explain the association between having children and the risk of developing diabetes later in life [1–3]. Pregnancy induces a state of insulin resistance in peripheral tissues which, in predisposed women, may be severe enough to lead to gestational diabetes, a condition that is strongly related to the risk of developing diabetes mellitus in the future [3, 22]. While glucose homoeostasis is restored to non-pregnancy levels shortly after delivery, it is unclear whether repeated exposure to insulin resistance has pathological perturbations many years after childbirth. The Coronary Artery Risk Development in Young Adults (CARDIA) study of 2,408 US women aged 18–30, however, showed that only women with gestational diabetes, and not those with normal glucose tolerance during pregnancy, were at a higher risk of type 2 diabetes compared with nulliparous women [23]. This does indicate that gestational diabetes reveals underlying susceptibility, and hence emphasises the importance of timely dietary, lifestyle and pharmacological interventions that might prevent or delay the onset of type 2 diabetes in affected women [3, 24]. Childbearing is also associated with gestational weight gain, changes in body fat distribution, and postpartum weight retention; alterations which all also could increase the risk of diabetes later in life. Nevertheless, in the present study, which excluded all those with self-reported and screen-detected diabetes, women with a higher number of children did not tend to have higher levels of BMI, nor were there discernible differences in the association at different levels of BMI. Instead, our study shows, for the first-time, that men with a high number of children are also at increased risk of diabetes, similar in magnitude to that in women. As it is difficult to imagine how physiological mechanisms could increase the risk of diabetes in men with a large number of children, the present study findings suggest that an interplay of social, cultural or psychological factors associated with raising children is more likely to underlie the findings in women than long-term diabetogenic consequences of pregnancy alone. For example, it is conceivable that financial, physical and mental pressures are greater in larger families than in smaller families, possibly resulting in reduced physical activity, more stress and increased intake of cheaper and unhealthier foods [25–27]. Greater support in achieving and maintaining healthy lifestyle and dietary habits might help in reducing the risk of diabetes seen in those with larger families. The finding of a potentially stronger effect of a greater number of children among individuals with hypertension compared with those without adds to the importance of enhanced blood pressure control among individuals with larger families.

Apart from providing the first comparative analyses of the association between number of children and risk of diabetes in women and men simultaneously, the present study benefits from a large sample size and detailed information collected on a wide range of socioeconomic, lifestyle and reproductive factors, which allowed for an in-depth examination of the association in both men and women. Our findings were largely robust for adjustment for several demographic, socioeconomic, physiological and lifestyle characteristics, some of which could act both as confounders and as effect mediators. For example, it is plausible that overweight individuals are less likely to conceive than individuals with a normal weight, but also that individuals with larger families are more likely to become overweight compared with those with smaller families. Since adjustment for effect mediators generally attenuates the effect estimates, our results might be conservative and underestimate the true association between parenthood and the risk of diabetes.

This study is not without limitations. First, even though our analyses excluded all those with self-reported and screen-detected diabetes, diabetes can remain asymptomatic and undiagnosed for years. It is therefore expected that we have missed individuals with incident diabetes. However, since the percentage of individuals with screen-detected diabetes at study baseline was similar between men and women (2.7% vs 2.8%) (ESM Table 2), it is conceivable that new onset of undiagnosed diabetes during follow-up is likely to have affected women and men to a similar extent, leading to an underestimate of the associations in both sexes. Second, while women with gestational diabetes are at a substantially increased risk of developing diabetes later in life [22], data on gestational diabetes were not available in the present study. Third, the reasons for having children or for being childless were unknown. Such information would have helped in reducing the amount of residual confounding, and in further examining potential mechanisms for the association between having children and the risk of diabetes. For example, the higher risk of diabetes among childless individuals could be due to health factors, such as overweight or polycystic ovary syndrome, that not only result in fertility problems but also increase the risk of diabetes. Although there was no material impact of adjustment for socioeconomic, physical and lifestyle factors on our findings, residual confounding by other factors, such as social or psychological determinants, cannot be excluded. Future studies with repeated measures of factors related to diabetes and other chronic conditions linked to number of children could help to better understand the mechanisms underlying the long-term health effects of childbearing and childrearing.

In conclusion, the present study demonstrates that, in a Chinese population, childlessness and a greater number of children are associated with an increased risk of diabetes in both women and men. Although the biological factors of parenthood and childbearing may mediate some of the effects, the similarity in the relationship between women and men suggests that social, cultural or psychological factors related to childrearing may be more important contributors.

## Electronic supplementary material

Below is the link to the electronic supplementary material.

ESM (PDF 155 kb)

## Abbreviations

CDC: Center for Disease Control and Prevention
CKB: China Kadoorie Biobank
